# Nucleosomes Are Stably Evicted from Enhancers but Not Promoters upon Induction of Certain Pro-Inflammatory Genes in Mouse Macrophages

**DOI:** 10.1371/journal.pone.0093971

**Published:** 2014-04-04

**Authors:** Alison Gjidoda, Mohita Tagore, Michael J. McAndrew, Alexander Woods, Monique Floer

**Affiliations:** Department of Biochemistry and Molecular Biology, Michigan State University, East Lansing, Michigan, United States of America; University of Crete, Greece

## Abstract

Chromatin is thought to act as a barrier for binding of *cis*-regulatory transcription factors (TFs) to their sites on DNA and recruitment of the transcriptional machinery. Here we have analyzed changes in nucleosome occupancy at the enhancers as well as at the promoters of three pro-inflammatory genes when they are induced by bacterial lipopolysaccharides (LPS) in primary mouse macrophages. We find that nucleosomes are removed from the distal enhancers of IL12B and IL1A, as well as from the distal and proximal enhancers of IFNB1, and that clearance of enhancers correlates with binding of various *cis*-regulatory TFs. We further show that for IFNB1 the degree of nucleosome removal correlates well with the level of induction of the gene under different conditions. Surprisingly, we find that nucleosome occupancy at the promoters of IL12B and IL1A does not change significantly when the genes are induced, and that a considerably fraction of the cells is occupied by nucleosomes at any given time. We hypothesize that competing nucleosomes at the promoters of IL12B and IL1A may play a role in limiting the size of transcriptional bursts in individual cells, which may be important for controlling cytokine production in a population of immune cells.

## Introduction

Genome-wide studies in *S. cerevisiae* have indicated that promoter regions are relatively depleted of nucleosomes compared to the surrounding regions [1], [2], [3]. Where it has been analyzed, for example at the *PHO5* and *GAL1/10* genes of yeast, it was found that removal of promoter nucleosomes is required for gene induction and is mediated by nucleosome remodelers (*e.g*. the Swi/Snf complex) that are recruited to these regions by specific TFs [4], [5]. At the *GAL1/10* promoters these nucleosomal sites are only lowly occupied prior to induction and low promoter nucleosome occupancy is at least partly determined by the underlying DNA-sequence and facilitates rapid nucleosome removal when the inducer galactose is added [6]. These studies have suggested that transcriptional regulatory regions have to be nucleosome-free to allow binding of *cis*-regulatory TFs and the transcriptional machinery. However, at least at one site of binding of a transcriptional activator, the *UASg* of the *GAL1/10* locus, it was shown that the consensus site-containing piece of DNA is part of an, albeit unusual, nucleosome that apparently accommodates activator binding on its surface [7]. Genome-wide studies in mammalian systems have similarly suggested that promoters are relatively depleted of nucleosomes [8], [9] and a recent study that analyzed the constitutively expressed KIT gene in mast cells showed that the promoter was nucleosome-free in this cell-type but not in others [10]. In addition, studies at many different genes in various cell-types that used changes in sensitivity of chromatin to the enzyme micrococcal nuclease (MNase), to Dnase I or to restriction enzymes, found that chromatin architecture was altered at promoters and enhancers when these genes were expressed indicating that nucleosomes are remodeled at these sites (see for example [11], [12]). In one well-studied example of an inducible gene, human interferon β, it was found that the promoter was cleared of nucleosomes upon viral induction, which led to clearing of the TATA-box [13]. The interferon β gene contains a promoter proximal enhancer, which forms an enhanceosome [14], and this close proximity of TF-binding sites to the transcriptional start site (TSS) resembles the typical gene architecture in yeast where TF-binding sites are usually within 500 bp of the TSS. However, other mammalian genes are often regulated by distal enhancer elements that can be thousands of base pairs away (for a recent review see [15]), and are thought to be brought in contact with the promoter by DNA-looping (for an example see [16]). This separation of enhancers and promoters at many mammalian genes prompted us to investigate the changes in nucleosome binding associated with either transcriptional regulatory element upon gene induction. We have used a quantitative assay to analyze changes in nucleosome occupancy at enhancers and promoters of three pro-inflammatory cytokines – IL1A, IL12B and IFNB1 - upon their induction by LPS in primary mouse macrophages. The assay uses a wide range of MNase concentrations and detects the distinct digestion rates of the same segment of DNA, when it is naked or associated with a nucleosome, which allows us to derive the fractional occupancy of a genomic region by a nucleosome [4].

Pro-inflammatory cytokines are expressed by macrophages as part of the innate immune response to various pathogens (for review see [17]) and requires the action of three main TFs, NFκB, AP1 and IRF3/7 [18]. Binding sites for these TFs are found in the regulatory elements of many pro-inflammatory genes [19], [20]. In addition to these signal-induced TFs at least two lineage-specific TFs, PU.1 and C/EBPβ, are required for macrophage differentiation and expression of certain pro-inflammatory genes [21], [22], [23], [24]. Both of these TFs have been found to be associated with regulatory elements of many genes even prior to their induction in macrophages [19], [20], [25]. The promoter proximal enhancer of IFNB1 is conserved in mice [26], but mouse IFNB1 was recently shown to also be regulated by a distal enhancer located 6 kb downstream of its TSS [27]. This region was found to also bind the *cis*-regulatory TF XBP when IFNB1 was induced by LPS and thapsigargin (TPG), an inducer of ER-stress that enhances expression of certain pro-inflammatory cytokines through the action of XBP. Furthermore, a minimal region of 305 bp that encompasses consensus-sites for XBP and IRF3 was shown to enhance transcription of a reporter gene confirming this region as a *bona fide* enhancer. Similar studies of the IL12B gene performed mostly by Stephen Smale's laboratory identified a distal enhancer located 10 kb upstream of its TSS [28]. This distal enhancer was shown to play a role in LPS induction of IL12B and was further found to strongly enhance IL12B expression in reporter assays that mimic the nucleosome environment found at the endogenous gene [28]. The distal enhancers of IL12B and IFNB1 were also classified as enhancers in two recent genome-wide studies [19], [20] that identified thousands of putative enhancers including a region located 10 kb upstream of the IL1A gene, which we have included in our studies as a putative enhancer for IL1A.

We find that nucleosomes in the distal enhancers of IL12B, IL1A and IFNB1 are rapidly evicted when the genes are induced. Nucleosomes are also removed from the proximal enhancer of IFNB1, which leads to clearance of the adjacent TATA-box and TSS as had been described for the human gene [13]. In addition, we show that nucleosome-depletion correlates with binding of *cis*-regulatory TFs and the co-activator p300 to the distal enhancers of all three genes as well as to the proximal enhancer of IFNB1. Surprisingly, we find nucleosomes at the IL12B and IL1A promoters in a large fraction of the population of cells under inducing conditions. Furthermore, we find that promoter nucleosomes around the TSSs of these genes become associated with histone modifications found at active promoters (H3K4me3 and H3K27ac). Our results indicate that promoter nucleosomes are not stably evicted but instead are bound to a fraction of promoters in the population of cells at any given time. Furthermore, we find that PolII and TBP are only associated with nucleosome-free promoters and we discuss the potential role of competing nucleosomes at the promoters of these cytokine genes in limiting their expression in a population of immune cells.

## Materials and Methods

### Ethics statement

Procedures to obtain primary cells from mice were performed under IACUC oversight (#07/12-113-00).

### Cell isolation and culture

Primary cells where isolated from 8–12 week old C57BL/6 mice (NCI). Bone marrow derived macrophages (BMDMs) were generated as described [29] and grown in BMDM medium (60% IMDM medium (Gibco), 30% conditioned medium from L-929 fibroblasts, 10% FBS, 0.1 mM non-essential amino acids, 1 mM sodium pyruvate and 1X penicillin-streptomycin. LPS induction was performed by adding 1 μg/ml LPS from *E. coli* strain EH100 (Ra mutant)(Sigma) to serum-starved BMDMs for the indicated times. Serum starvation was done by growth of cells in incomplete IMDM medium for 1 h. Other inducers were ISD (interferon stimulatory DNA) derived from *Listeria monocytogenes*; poly(I:C), synthetic dsRNA that acts as a TLR3 agonist; and poly(dA:dT), a synthetic analog of B-DNA (all obtained from Invivogen). 1 μg/ml of either of these inducers was given to BMDMs by transfection with Lipofectamine 2000 (Invitrogen) in an equal volume mixture [30]. Where indicated thapsigargin (Sigma) was added at 1 μM for 1 h to serum-starved cells prior to LPS addition [27]. Splenic B-cells were isolated by negative selection with CD43 antibody-coupled Dynabeads according to the instructions of the manufacturer (Life Technologies), with an additional red blood lysis step using lysis buffer (Sigma). For LPS induction B-cells were grown in B-IMDM medium (IMDM medium (Gibco), containing 55 μM 2-Mercaptoethanol and 2 mM L-glutamine) for 1.5 h prior to LPS addition for the indicated times. RAW264.7 cells were grown in DMEM medium (Gibco) containing 10% FBS and 1X penicillin-streptomycin.

### mRNA determination

Total RNA was isolated from BMDMs or B-cells using Trizol (Invitrogen/Lifetech). In brief, Trizol was added to cells growing in culture, and Trizol lysates were collected. 400 μl of chloroform was added per 1 ml Trizol lysate, the aequous phase was extracted, 170 μl isopropanol was added and the mixture was further purified on ReliaPrep RNA Cell Miniprep System columns according to the manufacturer's protocol (Promega). RNA was converted into cDNA according to the protocol described [31] except that High Capacity Reverse Transcriptase was used (Invitrogen/Lifetech) and analyzed by qRT-PCR with specific primer pairs. Primers used can be given upon request.

### Chromatin immunoprecipitation

Chromatin from 5×10^6^ cells per antibody that had been cross-linked with 0.5% formaldehyde for 10 min was isolated by sonication with a Branson sonifier (10 pulses of 10″ at setting 4) in Lysis buffer (50 mM Hepes-KOH, pH 7.5, 1% TritonX-100, 0.1% SDS) and centrifugation for 10′ at 21,000×*g*. To increase the resolution of ChIP experiments when detecting histones or histone modifications, and to differentiate nucleosome binding from PolII and TBP binding, the isolated chromatin was digested with 0.5 or 1 U MNase (NEB) for 1 h 30′ in the presence of 0.15 mM CaCl_2_, and the digestion reaction was stopped by addition of 20 mM EDTA. Digested chromatin was diluted 3-fold with High Salt ChIP buffer (10 mM Tris-HCl, pH 8, 400 mM NaCl, 1% TritonX-100, 2 mM EDTA, Complete protease inhibitors w/o EDTA (Roche)) to yield 600 μl total volume and incubated overnight at 4°C with either 5 μl of anti-H3 (39163, Active Motif, concentration is not known), 4 μg of anti-H2A.Z (ab4174; Abcam), 1 μg of anti-H3K4me1 (ab8895; Abcam), 1 μg of anti-H3K4me3 (ab8580; Abcam) or 1 μg of H3K27ac (ab4729; Abcam). For all other ChIP experiments isolated chromatin was directly diluted with High Salt ChIP buffer and incubated with either 1 μg of anti-PolI antibody (sc-56767), 6 μg anti-TBP (sc-204), 4 μg anti-PU.1 (sc-352), 4 μg anti-C/EBPβ (sc-150), 6 μg anti-NFκB (sc-372), 5 μg anti-c-Jun (sc-45), 6 μg anti-p300 (sc-585) or 10 μg anti-IRF3 (sc-9082) all from Santa Cruz Biotechnologies. 20 μl of Protein A/G magnetic beads (Pierce) were added to the reaction and incubated at 4°C for 2 h. Beads were washed with 280 μl each of TSE buffer (20 mM Tris pH 8.0, 0.1% SDS, 1% TritonX-100, 2 mM EDTA), TSE250 (TSE buffer, 250 mM NaCl) and TSE500 (TSE buffer, 500 mM NaCl), Wash buffer III (10 mM Tris pH 8.5, 0.25 M LiCl, 1% NP-40/Igepal, 1% deoxycholate, 1 mM EDTA) and TE (10 mM Tris-HCl pH 8.0, 1 mM EDTA) all containing Complete protease inhibitors. Antibody complexes were eluted from the beads with 2×100 μl Elution buffer (0.1 M NaHCO_3_, 1% SDS) by incubation for 30′ (and 10′) at 55°C. Eluates were combined and the cross-link was reversed by incubation at 65°C for 4 h. DNA was purified using a Qiagen PCR purification kit, and analyzed on a Lightcycler 480 (Roche) using primer pairs in the regions indicated. Sequences of the primers used can be given upon request.

### Quantitative nucleosome occupancy assay

The assay was performed essentially as described in [4] with certain modifications. Cross-linked chromatin from 1 to 3×10^7^ cells isolated as described for ChIP experiments was incubated in Lysis buffer containing 140 mM sodium chloride with 22 increasing concentrations of MNase (0.001179 U to 20 U, NEB) in the presence of 0.15 mM CaCl_2_ for 1 h 30′. DNA was purified as described and quantified using a Roche Lightcycler 480. Digestion data was analyzed using two-state exponential curve-fitting as described [4]. Data was normalized to several genomic locations, including a region in the promoter of KIT [10] that was highly protected and a region in the ORF of RPL4. The data was displayed in the IGV genome browser v2.3 [32] and overlays of nucleosome occupancy during a timecourse of LPS induction were created from IGV tracks using Adobe Photoshop.

### Genomic DNA isolation

Genomic DNA was isolated from RAW264.7 macrophages as described [33] and DNA standard curves were created using a 1/3 fold dilution series with the highest concentration yielding qRT-PCR amplification at around cycle 20 for the majority of primer pairs.

### qRT-PCR

DNA and cDNA was quantified on a Lightcycler 480 (Roche) as described [4] with the following modifications. Primers were designed using the program PCRtiler [34]. To verify that only a single amplicon was produced by each primer pair and no primer dimers were formed a T_m_-curve was performed as a quality control for each primer pair at the end of each qRT-PCR run. We also found that addition of 1.5% PEG400 (Fluka) to the qRT-PCR reaction greatly enhanced performance for many primer pairs and led to a greater linear range of the qRT-PCR measurements.

## Results

### Nucleosome occupancy at the IL12B enhancer and promoter upon LPS induction

**Figure 1:**
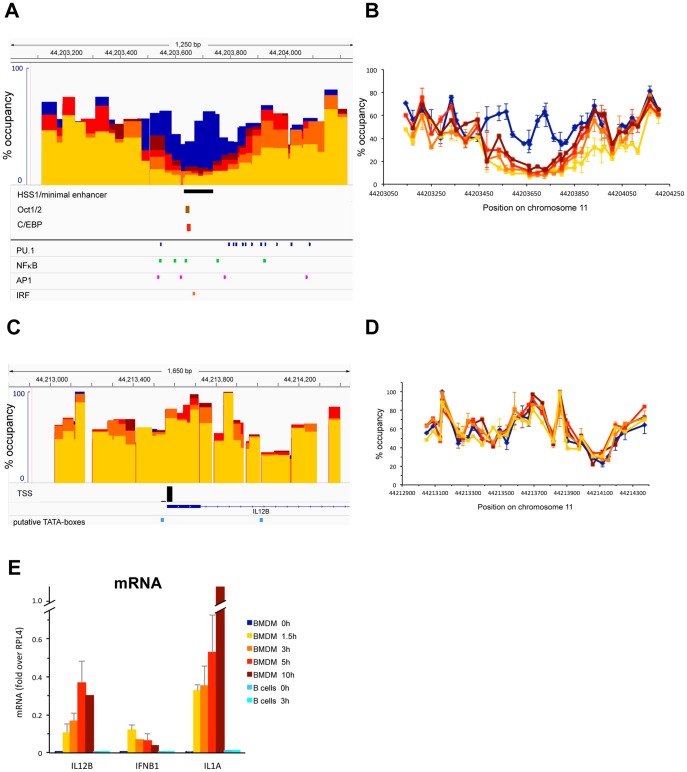
Changes in nucleosome occupancy upon LPS induction at a distal enhancer and the promoter of IL12B. (A and B), Nucleosome occupancy at an enhancer 10 kb upstream of the TSS of IL12B in BMDMs was analyzed before induction (blue bars and lines), and after 1.5 h (yellow), 3 h (orange), 5 h (light red) and 10 h (dark red) of growth of cells in the presence of 1 μg/ml LPS, using the assay described in [4] with modifications detailed in the Materials and Methods. In brief, occupancy was measured by determining the sensitivity of cross-linked chromatin to a wide range of MNase. Digestion data for each genomic location analyzed by qRT-PCR with specific primer pairs was fitted to two-state exponential decay functions and the percentage of DNA in the population of cells found to be protected against MNase by binding of a nucleosome is indicated on the y-axis. In panel (A) each overlapping colored bar represents the length of the amplicon measured. The minimal enhancer that was shown by Zhou et al. to contain the LPS-inducible DNaseI hypersensitive site HSS1 as well as consensus-sites for Oct1/2 and C/EBPβ is indicated by the black bar [28]. Consensus-sites for PU.1, NFκB, AP1 and IRF identified using ConSite are indicated. In panel (B) nucleosome occupancy at the midpoint of each amplicon measured by the experiment performed in panel (A) is indicated by a dot, with error bars showing the SEM of at least two independent measurements (10 h was measured only once). (C and D), BMDMs were induced as described in (A) and nucleosome occupancy in a region surrounding the TSS of IL12B was determined. The data is displayed as in panels (A) and (B) respectively. The black bar below the data in (C) indicates the TSS [35] and the light blue bars indicate putative TATA-boxes predicted by ConSite. (E), Expression of IL12B, IFNB1 and IL1A in response to LPS. mRNA from BMDMs induced with LPS as in panel *A* as well as from splenic B-cells was isolated as described in the Materials and Methods, reverse transcribed and cDNA quantified by qRT-PCR. Data was normalized to a location in the ORF of the constitutively expressed RPL4 gene. The SEM of at least two independent measurements is indicated (10 h timepoint was measured only once).

Figure 1A and B shows an analysis of nucleosome occupancy in a 1.2 kb region encompassing the 10 kb upstream enhancer of IL12B [28] at different timepoints during LPS induction of primary bone-marrow derived macrophages (BMDMs) using the assay described [4]. Prior to induction (blue bars and lines) nucleosomes in the IL12B enhancer were relatively well positioned and occupied their sites in around 60% of the population of cells. 1.5 h after LPS addition (yellow) two nucleosomes in the center of the enhancer had been removed. The 5–10% occupancy we detected upon clearance of these nucleosomes is within the accuracy of our assay and we conclude that this region was largely nucleosome-free after 1.5 h. The central nucleosomal position, which remained cleared upon prolonged incubation with LPS up to 10 h (dark red), coincides with a region that was shown by Zhou et al. to become hypersensitive to Dnase I upon LPS induction (see the black bar underneath panel A [28]). We found that the flanking nucleosomes were partially re-formed as induction progressed and after 5 h of induction the nucleosome to the left was again occupied in 30% of the population (light red). The nucleosome to the right was partially removed after 1.5 h (30–40%) and regained 60% occupancy after 5 h (light red). We monitored expression of the associated IL12B gene by measuring mRNA levels during the 10 h timecourse (Figure 1E). IL12B mRNA was detected as early as 1.5 h after LPS addition, and levels increased for up to 5 h, after which IL12B mRNA production reached steady-state levels. Figure 1E also shows mRNA levels upon LPS induction of IFNB1 and IL1A.

Figure 1C and D shows nucleosome occupancy at the IL12B promoter including a region 600 bp upstream and 800 bp downstream of the TSS. Surprisingly, we did not find any changes in nucleosome occupancy upon LPS induction over the 10 h timecourse of LPS induction (compare blue bars and lines to increasing shades of red). The region surrounding the TSS was more highly occupied by nucleosomes than the enhancer prior to induction and nucleosomes were less well positioned than in the IL12B enhancer. We found that the region directly upstream of the TSS was occupied in about 60% of the population and this region was flanked by more highly occupied nucleosomes (around 90%). A TATAA-sequence that we identified 28 bp upstream of the TSS (light blue box in C) as well as the TSS itself was found at the edge of the highly occupied nucleosome. We found that a region 400 bp downstream of the TSS that contains a TATAT-sequence was relatively lowly occupied by nucleosomes prior to induction (20–30%), which had initially suggested to us that this downstream region might function to assemble a pre-initiation complex. However, a previous search for TSSs that used CAGE-analysis to detect capped mRNAs had not found any transcription starting from this downstream region, but had instead confirmed the annotated TSS for IL12B [35]. We therefore conclude that the upstream TATAA-sequence is used to assemble a PIC. This conclusion was confirmed by our subsequent ChIP analysis, which detected PolII and TBP binding at this site (see Figure 3).

### Changes in nucleosome occupancy at the transcriptional regulatory regions IL1A

**Figure 2:**
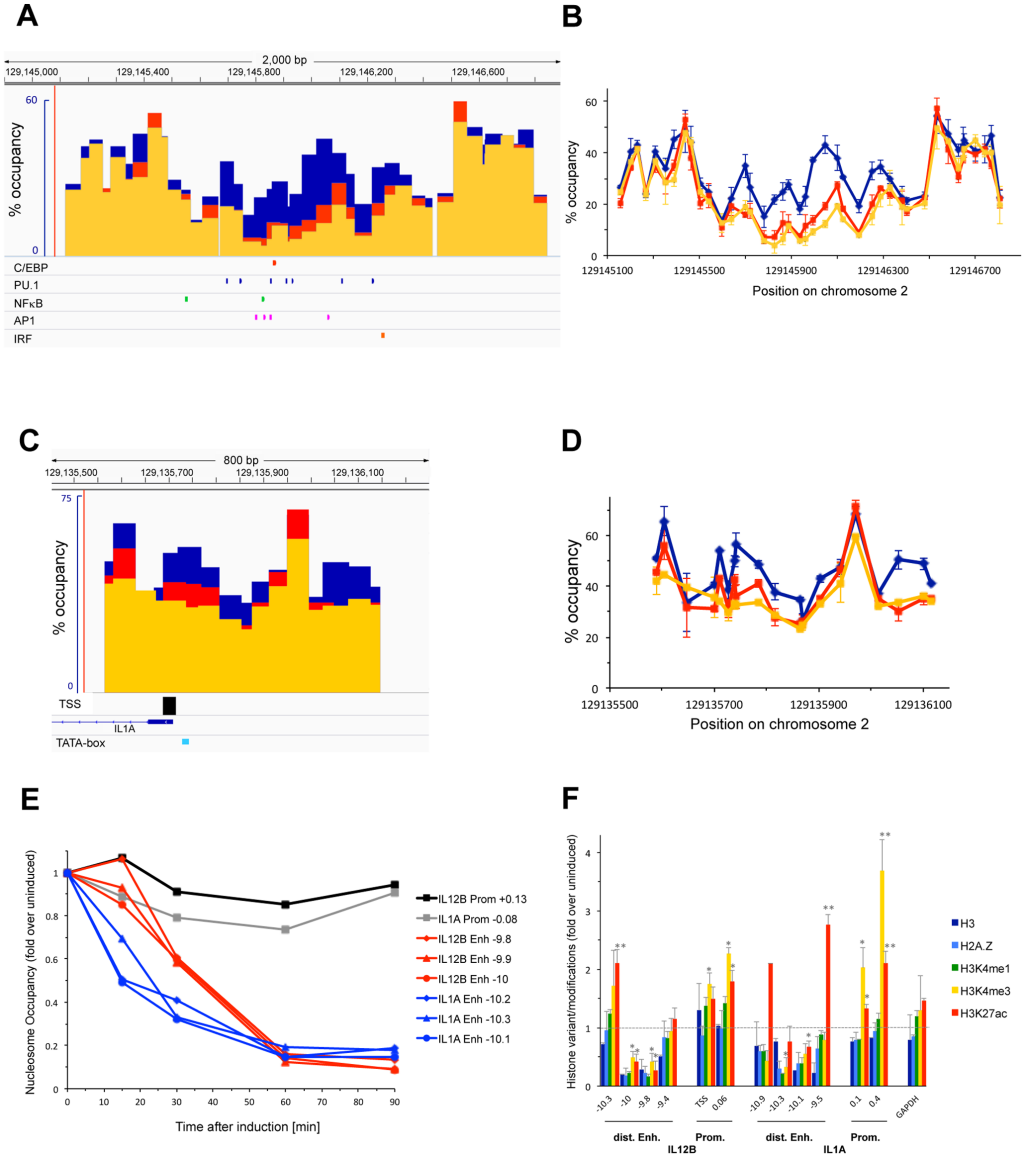
Changes in nucleosome occupancy upon LPS induction at a putative distal enhancer and promoter of IL1A, kinetics of nucleosome removal, and changes in histone modifications. (A and B), Nucleosome occupancy at a putative enhancer 10 kb upstream of the TSS of IL1A was determined in BMDMs prior to (blue bars and lines) and upon 1.5 h (yellow) or 3 h (red) induction with 1 μg/ml LPS as described in the legend of Figure 1. ConSite predicted consensus sites for PU.1, C/EBP, IRF, AP1 and NFκB are indicated. (C and D), Nucleosome occupancy at the promoter of IL1A was determined as described in panel (A) in a region surrounding the TSS of IL1A. The TSS (black bar) [35] and a putative TATA-box (blue bar) is indicated in panel (C). (E), Kinetics of nucleosome removal at IL1A and IL12B. BMDMs were induced with LPS for 15′, 30′, 60′ and 90′, and nucleosome occupancy was analyzed as described in (A). Nucleosome removal at three locations in the distal enhancer of IL12B (red lines) and in the enhancer of IL1A (blue lines) is shown. The data is displayed as the fold change in nucleosome occupancy over the levels found before induction at each location. The absence of changes in nucleosome occupancy at the TSS of IL12B (black line) and of IL1A (gray line) is shown for comparison. (F), ChIP experiments with antibodies against H3 (dark blue bars), H2A.Z (light blue), H3K4me1 (green), H3K4me3 (yellow) and H3K27ac (red) were performed as described in the Materials and Methods. For these experiments cross-linked chromatin was lightly digested with MNase before incubation with the respective antibodies to increase resolution of the ChIP signal and the data was normalized to a region in the ORF of RPL4. Changes upon LPS induction in histone binding and histone modifications at the enhancers and promoters of IL12B and IL1A as well as at a control region in the GAPDH pseudo gene are shown as fold over levels found before induction. For H3K27ac the changes 1.5 h after LPS induction, and for all other histone variants and modifications the changes after 3 h of induction are shown. The error bars show the SEM of at least 3 independent experiments. Statistical significance of the changes in H3K4me3 and H3K27ac upon LPS induction compared to levels found prior to induction determined by *Student's* T-tests is indicated (*P<0.05; **P<0.01).

Figure 2 shows an analysis of nucleosome occupancy at a putative enhancer 10 kb upstream (panel A and B) and around the TSS (panel C and D) of the IL1A gene before (blue bars and lines) and upon induction of macrophages with LPS for 1.5 h (yellow) and 3 h (red). Similar to our findings at the IL12B enhancer we found that the putative IL1A enhancer was depleted of nucleosomes 1.5 h after LPS addition. This region encompasses 3–4 nucleosomes, which were occupied in 40–60% of the population prior to induction. The center of this region became essentially nucleosome free (5–10%) and remained so even after prolonged LPS induction (3 h, red bars and lines in panels A and B). The three nucleosomes flanking this region became partially depleted upon LPS induction (20–30% occupancy after 1.5h) and occupancy of these flanking nucleosomes increased slightly upon prolonged LPS induction similar to what we had found at the IL12B enhancer (compare yellow and red bars and lines in Figure 2A–D).

Panels C and D of Figure 2 show that the promoter of IL1A was not cleared of nucleosomes upon induction. We found that prior to LPS induction the IL1A promoter was less occupied by nucleosomes than the IL12B promoter. Thus, a nucleosome that incorporates the TSS and TATAA-sequence of IL1A was occupied in about 55% of the population of cells before induction. Upon LPS induction nucleosome occupancy at the TSS decreased somewhat (35% after 1.5 h, yellow bars and lines) and then increased again as LPS induction progressed (45% at 3 h, red). As for IL12B, the annotated TSS was confirmed as the major TSS for IL1A by Carninci and colleagues [35] and is indicated by the black bar underneath panel C. As shown in Figure 1E we found that IL1A mRNA levels increased during a 10 h course of LPS induction, suggesting that IL1A transcription is sustained over this time period. We were not able to determine nucleosome occupancy in a region 100–400 bp downstream of the TSS of IL1A, since this region consists almost entirely of CTT or CCT repeats and is resistant to qPCR.

### Timing of enhancer nucleosome removal

To determine the earliest timepoint of nucleosome removal at the distal enhancers of IL12B and IL1A we analyzed nucleosome occupancy in the centers of the two enhancers 15′, 30′, 60′ and 90′ after LPS induction. Figure 2E shows that the IL1A enhancer was significantly depleted 15′ after LPS induction (blue lines), whereas no nucleosomes had been removed at the IL12B enhancer at this early timepoint (red lines). Figure 2E indicates the fold change of nucleosome removal over the levels found before induction and nucleosome occupancy before induction was similar at the three representative locations in each enhancer. Nucleosome depletion at the IL1A enhancer was close to completion after 30′, while depletion at the IL12B enhancer had only reached 50%. After 1 h both enhancers had reached their maximal levels of nucleosome depletion. Our results show that nucleosome removal at the IL1A enhancer occurs with faster kinetics than at the IL12B enhancer.

### Histone modifications at the promoters and enhancers of IL12B and IL1A

Figure 2F shows the results of ChIP experiments performed with various antibodies that detect histone H3, the histone variant H2A.Z as well as different modifications of residues in H3 upon induction of BMDMs with LPS. We first confirmed that nucleosomes are evicted from the enhancers of IL12B and IL1A but not the promoters using an antibody against H3. Figure 2F shows that the H3 signal decreased upon LPS induction only in the regions in the enhancers where nucleosomes were evicted (compare to Figure 1A and 2A). We found similar results using an antibody against H2A.Z at the enhancers and promoters of both genes, or with an antibody detecting H3K4me1, which was previously shown to be present at the enhancers prior to and upon LPS induction [19], [20]. Most importantly, we detected an increase in H3K4me3 and H3K27ac at the promoters of IL12B and IL1A upon induction. Both modifications have previously been shown to be associated with actively transcribed genes [36], [37] and to increase at the two genes we have investigated upon their induction [38].

### Binding of cis-regulatory TFs to the distal enhancers of IL12B and IL1A

The minimal enhancer of IL12B was previously shown to bind C/EBPβ and Oct1/2 upon induction and consensus-sites for these TFs were identified in this region [28]. We used the prediction program ConSite [39] to identify consensus-sites for other TFs involved in induction of pro-inflammatory genes in macrophages and found consensus-sites for PU.1, NFκB, AP1 and IRF3 in the region that becomes depleted upon induction (see Figure 1A). A similar survey of the putative enhancer of IL1A also detected consensus sites for PU.1, C/EBP, IRF3, AP1 and NFκB in the region that is depleted of nucleosomes upon LPS induction (see Figure 2A).

**Figure 3:**
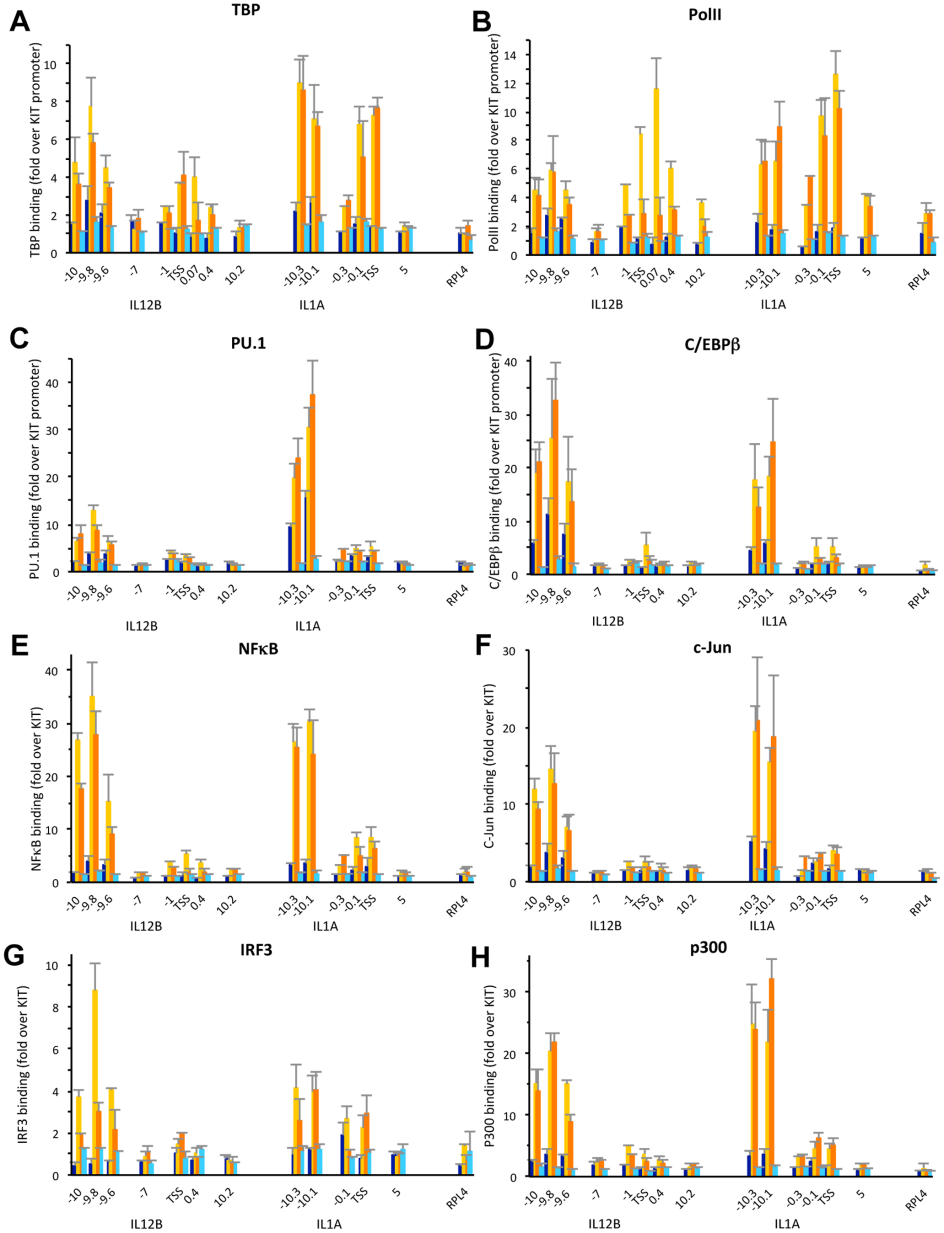
Binding of *cis*-regulatory TFs and recruitment of the transcriptional machinery to the regulatory regions of IL12B and IL1A upon LPS induction. (A–H), ChIP experiments in BMDMs before (dark blue bars), and upon 1.5 h (yellow) and 3 h (orange) of LPS induction as well as in splenic B-cells (light blue) were performed as described in the Materials and Methods using antibodies that recognize (A) TBP, (B) PolII, (C) PU.1, (D) C/EBPβ, (E) NFκB, (F) c-Jun, (G) IRF3 and (H) p300. Binding data was normalized to a location in the promoter of the KIT gene, and genomic locations in relation to the TSS of IL12B or IL1A are indicated on the x-axis in each panel. Binding to a control region in the RPL4 ORF is shown for comparison. Error bars indicate the SEM of at least three independent experiments. Statistical significance for binding in each region was determined by *Student's* T-tests performed for each regulatory region (see Table S1 for P-values).

To analyze binding of these TFs to the distal enhancers of IL12B and IL1A as well as recruitment of the transcriptional machinery to the enhancers and promoters we performed ChIP experiments in uninduced macrophages and cells induced for 1.5 and 3 h with LPS (Figure 3). We found that PolII and TBP were recruited to the TSS of both IL12B and IL1A upon induction (Figure 3A and B). We also found that similar amounts of PolII and TBP were recruited to the distal enhancers of both genes but not to a control region between the IL12B TSS and the distal enhancer (−7 kb). For these and all other ChIP experiments we used splenic B-cells as a control (light blue bars). The three genes we have investigated were not induced by LPS in B-cells (see Figure 1E, cyan bars) and no factor binding was detected (see Figure 3). We also determined binding of the macrophage-specific TFs PU.1 and C/EBPβ and confirmed their presence at the two distal enhancers before LPS induction (Figure 3C and D, dark blue bars)[19], [20]. Upon induction binding of both factors to the two distal enhancers increased significantly (compare yellow and orange to dark blue bars). We found similar results when we performed a ChIP experiment with an antibody for C/EBPα, indicating that both C/EBP isoforms are present (A.G. and M.F., data not shown). Furthermore, we detected binding of NFκB, c-Jun (a component of AP1) and IRF3 at the enhancers upon LPS induction (Figure 3E-G). The coactivator p300 was previously shown to be recruited upon LPS induction to the regions encompassing the IL12B as well as the putative IL1A enhancer [19], a finding we confirmed (Figure 3H). Each ChIP experiment was performed at least three times and error bars (SEM) are included. We determined the significance of the detected ChIP signals by performing *Student's* T-tests (Table S1). To obtain robust statistics we pooled all the measurements at the different loci in the enhancer or promoter regions of either gene from 3–4 independent experiments. Overall we find that binding of the *cis*-regulatory TFs and the co-activator p300 is significant in the enhancers, while binding of PolII and TBP is significant at both enhancers and promoters.

### IFNB1

**Figure 4:**
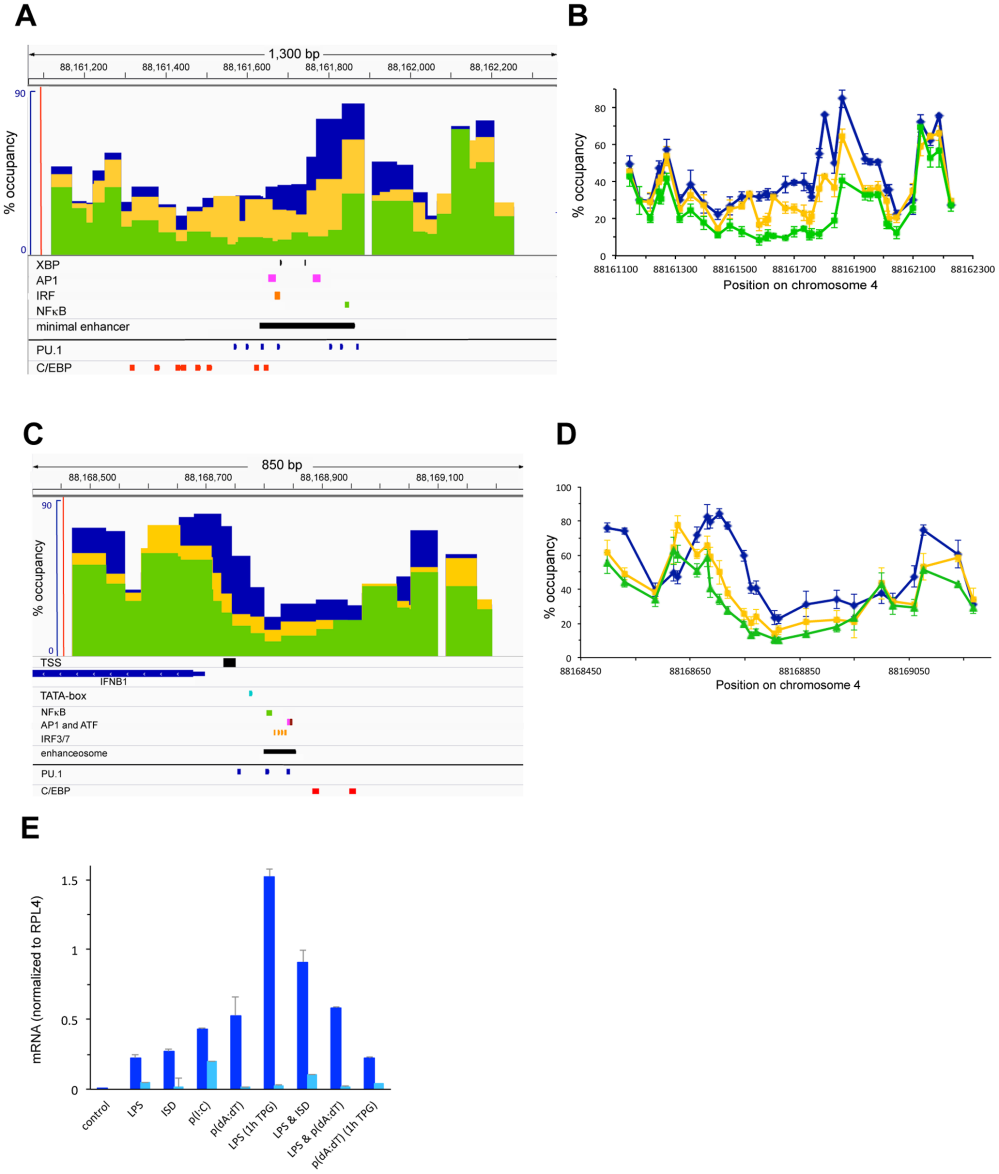
Nucleosome occupancy at the distal enhancer as well as at the proximal enhancer and the promoter of IFNB1 upon LPS and TPG induction. (A and B), Nucleosome occupancy was determined in BMDMs before induction (blue bars and lines), and upon induction with 1 μg/ml LPS for 1.5 h with (green) or without (yellow) pretreatment of cells with 1 μM TPG for 1 h. The minimal enhancer region (black bar) and binding sites for XBP, AP1, IRF3 and NFκB identified by [27] are shown in (A). ConSite predicted binding sites for PU.1 and C/EBP are indicated. (C and D), Nucleosome occupancy at the proximal enhancer and promoter of IFNB1 was determined as in panel *A* and analyzed in a region encompassing the proximal enhancer that is conserved in humans and has been shown to form an enhanceosome upon viral stimulation of HeLa cells [14], as well as in the 5′ region of the IFNB1 ORF. Conserved binding sites for NFκB, ATF, AP1 and IRF3/7 identified by [26] are indicated. ConSite-predicted consensus sites for PU.1 and C/EBP are also shown. *E*, Expression of IFNB1 upon stimulation with different inducers. BMDMs were induced for 3 h (dark blue) or 16 h (light blue) with 1 μg/ml of LPS, or 1 μg/ml of ISD, 1 μg/ml p(I:C), or 1 μg/ml p(dA:dT) added either alone or together with LPS as indicated. Where indicated cells were pre-treated with the ER-stress inducer TPG for 1 h prior to LPS induction. mRNA was isolated and quantified as described in the Materials and Methods. Data was normalized to the ORF of RPL4.

We found that IFNB1 was only moderately induced by LPS (Figure 1E), a result also reported by others [40]. To further increase induction, we treated macrophages with other inducers of this cytokine either alone or in addition to LPS (Figure 4E). As shown in Figure 4E we found transient induction of IFNB1 with various inducers (*i.e*. ISD, p(I:C), p(dA:dT)) either alone or in combination with LPS. However, the strongest increase in IFNB1 expression was seen when cells were pre-treated with the ER-stress inducer TPG prior to LPS induction (see also [27]). We therefore analyzed nucleosome occupancy at the regulatory regions of IFNB1 upon induction with LPS alone or after pretreatment with TPG. Figure 4A and B shows nucleosome occupancy at the enhancer 6 kb downstream of the TSS of IFNB1 before LPS induction (blue bars and lines) or 1.5 h after LPS induction with (green) or without (yellow) TPG pretreatment. We find that in resting macrophages the region encompassing the minimal enhancer region defined by Zeng et al. (black bar in Fig. 4A, taken from [27]) partially overlaps with a highly occupied nucleosome (80–90%). To the left of this highly occupied site nucleosome positions are less well defined and occupancy was found to be lower (around 40%). Nucleosome occupancy in this region only slightly decreased when cells were induced with LPS alone for 1.5 h. However, when cells were pretreated with TPG prior to LPS induction, nucleosomes were completely removed from the lowly occupied region (5–10% remaining) and partially from the highly occupied nucleosomal site. The region that was cleared of nucleosomes encompasses binding sites for the TPG-induced TF XBP, as well as for AP1 and IRF3 (see Fig. 4A)[27]. A binding site for NFκB is located in the region [27] that we find is highly occupied by a nucleosome before induction and becomes partially cleared upon induction. We also identified consensus-sites for PU.1 and C/EBP in the nucleosome-depleted region using ConSite (Figure 4A).

Figure 4C and D shows nucleosome occupancy at the promoter and the promoter proximal enhancer of IFNB1, which forms an enhanceosome upon induction (indicated by the black bar underneath Fig. 4C and taken from [26]), both prior to (blue bars and lines) and upon LPS induction of the gene with (green) and without (yellow) TPG pretreatment. We find that the enhanceosome is formed in a region that spans a linker region between two nucleosomes as has been described for the human gene. The nucleosome on the right was found to be lowly occupied (40%) and partly covered the enhancer. The nucleosome to the left was highly occupied (90%) and encompasses the TSS and TATAA-sequence of IFNB1. Upon LPS induction the region that forms an enhanceosome was partially cleared of nucleosomes (around 20%). Similar to our findings at the distal enhancer of IFNB1 we found that pretreatment of cells with TPG prior to LPS induction led to further depletion of nucleosomes at the proximal enhancer, which became essentially nucleosome-free in the presence of TPG and LPS (5–10%). The nucleosome to the right of the enhanceosome was partially depleted and the nucleosome to the left was shifted to a downstream position, which led to clearance of the TSS and TATAA-sequence as has been described for the human gene [13].

### TF binding to the distal and proximal enhancers of IFNB1

**Figure 5:**
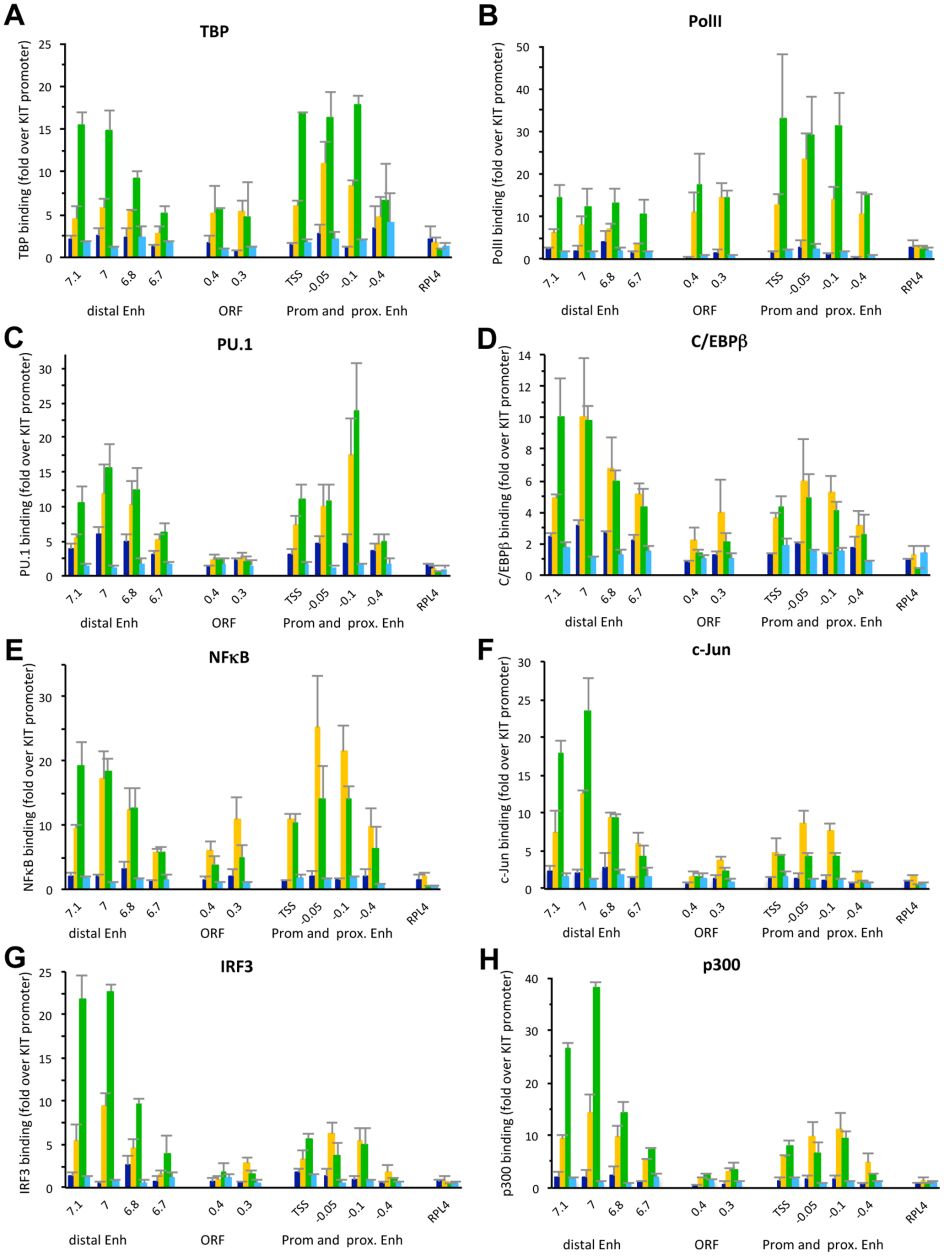
Binding of TFs and recruitment of the transcriptional machinery to the distal and proximal enhancers of IFNB1. (A–H), ChIP experiments were performed as described in the legend of Figure 3 in BMDMs before (dark blue), and upon 1.5 h LPS induction with (green) or without (yellow) pretreatment of cells with TPG, as well as in splenic B-cells (light blue). The antibodies used in each ChIP experiment are as in Figure 3 and are indicated. Error bars indicate the SEM of at least three independent experiments and statistical significance of binding of these factors to the different regions was determined by *Student's* T-tests (see Table S2).

To determine binding of *cis*-regulatory TFs and the transcriptional machinery to the distal as well as to the proximal enhancer and the promoter of IFNB1 upon induction of the gene we performed ChIP experiments. Figure 5 shows that all the factors tested were recruited to both the distal as well as to the proximal enhancer of IFNB1. Due to the proximity of the proximal enhancer to the promoter, including the TSS and TATAA-sequence, our ChIP experiments cannot distinguish binding to the promoter and promoter proximal enhancer. As shown in Figures 5A and B we found more binding of TBP and PolII to the proximal enhancer/promoter when cells were pretreated with TPG prior to LPS induction (compare green to yellow bars) in agreement with the increase in gene expression we observed (Figure 4E). As seen for the distal enhancers of IL12B and IL1A (Figure 3A and B) we also found binding of TBP and PolII to the distal enhancer of IFNB1 upon induction. Furthermore, we found that binding of PU.1 to the proximal and distal enhancer increased somewhat when cells were pretreated with TPG (panel C). C/EBPβ and NFκB binding did not increase significantly at the distal enhancer upon TPG treatment over levels seen when cells were treated with LPS alone and binding to the proximal enhancer was somewhat decreased. In contrast, we found a significant increase in binding of c-Jun, IRF3 and p300 to the distal enhancer upon TPG pretreatment, while binding to the proximal enhancer remained the same or decreased slightly (panels F–G). We hypothesize that complete nucleosome removal from the distal enhancer after pretreatment of cells with TPG prior to LPS induction (see Figure 4A and B) facilitated binding of the *cis*-regulatory TFs tested under these conditions. While the further increase in nucleosome removal upon pretreatment with TPG at the promoter proximal enhancer was less dramatic than at the distal enhancer, it correlated with increased binding of some TFs (PU.1) and increased recruitment of the transcriptional machinery.

### Binding of the transcriptional machinery to nucleosome-free IL12B and IL1A promoters

**Figure 6:**
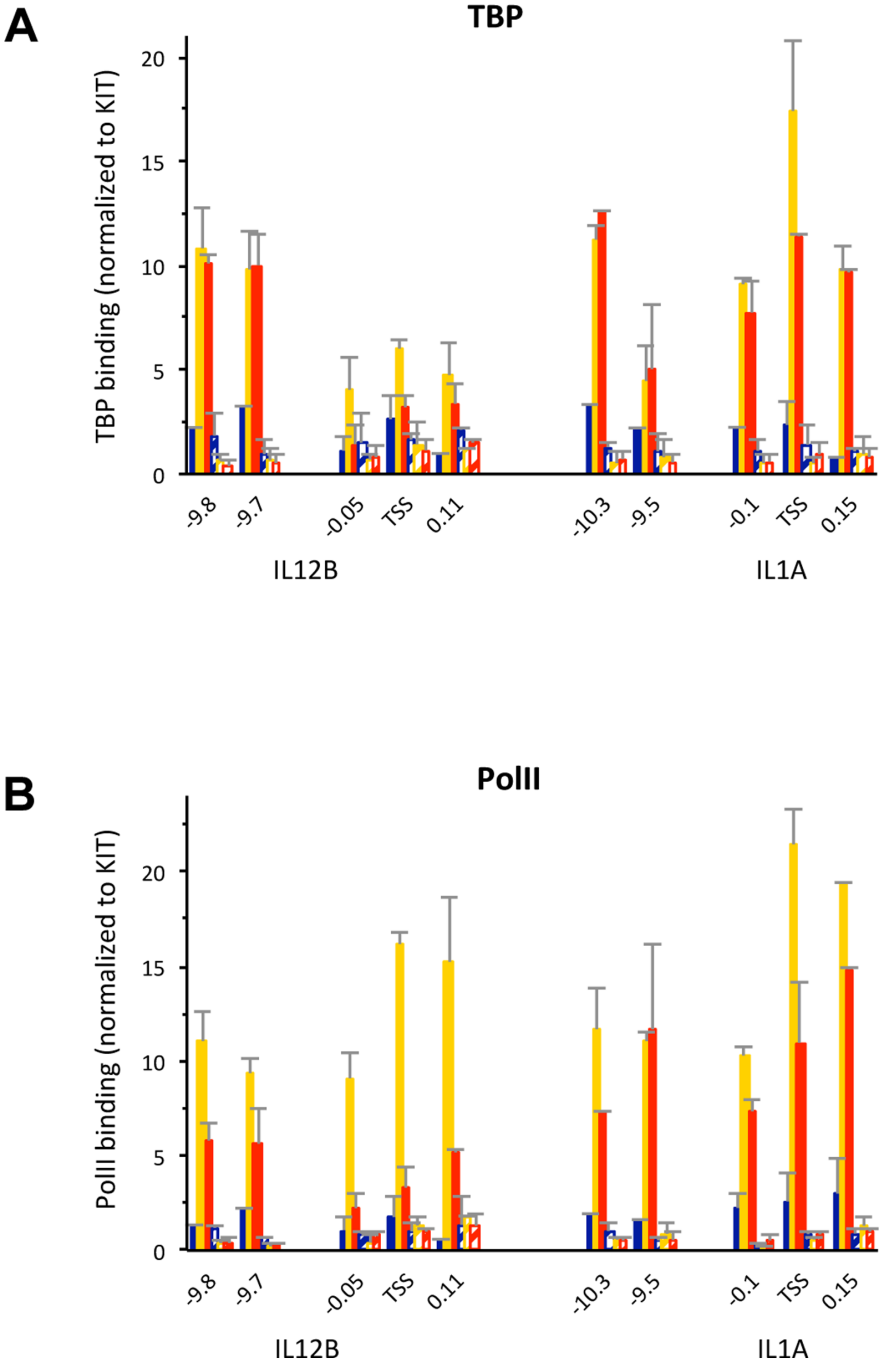
PolII and TBP binding in the fraction of IL12B and IL1A promoters in a population of induced BMDMs that is nucleosome-free. (A and B), ChIP experiments were performed as described in the legend of Figure 2F with antibodies that detect (A) PolII or (B) TBP in BMDMs before (dark blue bars), and upon 1.5 h (yellow) or 3 h (red) LPS induction. Cross-linked chromatin was either untreated (solid bars), or lightly digested with MNase (hatched bars) as described in the Materials and Methods. The data was normalized to a region in the KIT promoter and genomic locations are indicated. The experiment was performed twice and error bars indicating the SEM are shown.

To determine whether PolII and TBP might bind to the promoters of IL12B and IL1A in the presence of nucleosomes or whether the transcriptional machinery is only associated with the fraction of promoters that is nucleosome-free we performed the experiment shown in Figure 6. For this experiment we treated cross-linked chromatin with MNase prior to performing a ChIP experiment with antibodies detecting PolII or TBP. As seen in Figure 6 the PolII or TBP ChIP-signal was lost when chromatin was treated with MNase (compare solid to hatched bars). In contrast, H3, modified H3K4me3 or H3K27ac was resistant to pretreatment with MNase (see Figure 2F). This result indicates that only the fraction of the promoters that is nucleosome-free at any given time is associated with the transcriptional machinery.

## Discussion

Our analysis of nucleosome occupancy at the regulatory regions of three pro-inflammatory genes revealed that the distal enhancers of IL12B and IFNB1 were rapidly cleared of nucleosomes when the genes were induced. The regions that became nucleosome-free include the respective minimal regions that had been shown to have *bona fide* enhancer activity by previous studies (see Figure 1A and 4A)[27], [28]. We found similar removal of nucleosomes in a region 10 kb upstream of IL1A, which has been suggested to be a functional enhancer of IL1A (Figure 2A)[19]. In all three distal enhancers the nucleosome-free regions became associated with the TFs NFκB, AP1 (c-Jun) and IRF3 upon LPS induction, while binding of the macrophage-specific TFs PU.1 and C/EBPβ increased (see Figure 3 and 5). The presence of consensus-sites for these TFs was confirmed with the prediction program ConSite [39](Figure 1A, 2A, 4A). Together our data suggest that the enhancers of these pro-inflammatory genes have to be cleared of nucleosomes to allow binding of *cis*-regulatory TFs, although it remains to be determined whether binding occurs only to sites that become nucleosome-free or also to putative consensus-sites found in the surrounding regions (M.F., data not shown) that remain bound by nucleosomes. Future studies will show whether removal of nucleosomes from consensus-sites can be used as a criterion to distinguish functional binding-sites for specific *cis*-regulatory TFs in the genome from sites that remain associated with nucleosomes and may therefore not be accessible.

The most surprising result of our study was the finding that the promoters of IL12B and IL1A were not cleared of nucleosomes when the genes where expressed, while nucleosomes were rapidly removed from the associated distal enhancers. Thus, we found that the TSS of IL12B was occupied in about 70% of the population prior to induction and remained essentially unchanged, while the distal enhancer became nucleosome-free in about 90% of the population (see Figure 1). We found similar results at the distal enhancer and promoter of IL1A (Figure 2). The presence of nucleosomes at the promoters before and after LPS induction was further confirmed by our histone ChIP experiments (Figure 2F). In these experiments, we also detected an increase in H3K4 tri-methylation and H3K27 acetylation of the highly occupied promoter nucleosomes of IL12B and IL1A in agreement with previous lower resolution studies (Figure 2F, yellow and red bars)[19], [38]. Our finding that MNase treatment abolished the PolII and TBP ChIP-signal at the IL12B and IL1A promoters (Figure 6) strongly suggests that the transcriptional machinery is only associated with the fraction of promoters that is nucleosome-free at any given time. We speculate that in contrast to the stable eviction of nucleosomes at enhancers, which persisted over the timecourse of our induction experiment, nucleosomes may continuously re-associate with the promoters of IL12B and IL1A. This would allow only a fraction of the cells to form a PIC at any given time. This idea is in agreement with previous findings that expression of many inducible genes, including the genes we have analyzed, is highly stochastic [41], [42], [43], [44]. Another finding that supports the idea that a changing fraction of the population of cells expresses these genes at any given time, was the observation made by Smale and co-workers that expression of IL12B is not restricted to a clonal fraction of a population in a macrophage cell-line under inducing conditions [41]. We hypothesize that the presence of competing nucleosomes at the promoters of these cytokines may play a role in limiting the burst size of transcription from individual cells and thus the production of cytokines in the population. We further speculate that certain histone modifications might play a role in increasing nucleosome turnover at these promoters, a hypothesis that awaits experimental confirmation.

Our findings are in contrast to previous findings by Weinmann et al., which had suggested that a region about 200 to 330 bp upstream of the TSS of IL12B is nucleosome-free even prior to activation in macrophages (both in cell-lines and thioglycollate-elicited peritoneal macrophages) using sensitivity of chromatin to MNase followed by indirect end-labeling or ligation-mediated PCR to determine nucleosome binding [41]. These authors had also suggested that a region downstream of the putative nucleosome-free region contained a positioned nucleosome, which they proposed to harbor putative binding sites for NFκB (Rel) and C/EBP. Upon activation they found that this region became more sensitive to various restriction enzymes as well as to Dnase I [41], and they suggested that remodeling of the positioned nucleosome might facilitate binding of *cis*-regulatory TFs. We did not find significant binding of NFκB or C/EBPβ to this region upon LPS induction compared to the strong binding we found at the 10 kb upstream enhancer (see Figure 3). Nor did we find a nucleosome-free region in the IL12B promoter prior to induction even when we extended our analysis to include up to 1.5 kb upstream of the TSS of IL12B (Figure. 1 and A.G. and M.F., unpublished data). Our quantitative MNase sensitivity assay showed that upon induction there was no significant change in the level of nucleosome occupancy at the IL12B promoter in the population of cells (Figure 1), which was confirmed by histone ChIP experiments (Figure 2F). It is possible that our assay does not detect more subtle changes in nucleosome binding that might be induced by nucleosome remodeling and which may be detected by increased sensitivity of chromatin to certain restriction enzymes or Dnase I [41]. Furthermore, it is formally possible that macrophages derived from bone-marrow may be different from those derived from the peritoneum or from macrophage cell-lines.

IL1A contains additional regions between the 10 kb distal enhancer we have investigated and the TSS that become associated with TFs upon induction in dendritic cells [38]. This might suggest that additional enhancers may also control expression of IL1A in primary macrophages, and it remains to be seen whether nucleosomes are similarly evicted from such sites. The nucleosomes that are evicted from the distal enhancers of all the genes we have analyzed are only occupied in 40–60% of a population of resting macrophages, which is lower than the occupancies we found at, for example, the TSS of IL12B and IFNB1 (see Figure 1D, 4D). Our findings of moderate nucleosome occupancy at enhancers are in agreement with a previous study of an enhancer upstream of the KIT gene in mouse myeloid cells, where occupancy was found to be around 55% [10]. Whether this moderate level of nucleosome occupancy allows rapid induction of these and other genes remains to be determined. We also found significant [45]genes, while intervening regions (*e.g*. a region 7 kb upstream of the TSS of IL12B) showed no binding of these factors (see Figure 3A and B, 5A and B). This finding is in agreement with the presence of the transcriptional machinery at the enhancers other actively transcribed genes (see for example [46], [47]). It has been shown that DNA looping can bring distal enhancers into close proximity of promoters [16], [45], and it is therefore possible that we detected PolII and TBP at the enhancers merely as a result of DNA looping. However, our experiments showed clear enrichment of signal-induced TFs and the co-activator p300 at the distal enhancers of IL12B and IL1A with very little binding at the promoters (Figure 3C–H). These results indicate that our ChIP assay can distinguish between genomic locations that are contacted directly by *cis*-regulatory TFs and the general machinery, and those that may come into proximity of these factors only indirectly as a result of DNA looping. We therefore believe that PolII and TBP are directly recruited to the distal enhancers. Our results are in agreement with previous findings that many active enhancers are transcribed and produce short eRNAs [48], [49], but what the role of transcription initiating from such sites might be remains to be determined.

In contrast to our findings at the IL1A and IL12B promoters we found that the TATAA-sequence in the IFNB1 promoter was cleared of nucleosomes upon induction in primary mouse macrophages as had been described for the IFNB1 promoter in human cells (Figure 4C and D)[13]. IFNB1 contains a conserved proximal enhancer, which became associated with all the TFs we tested as well as with the co-activator p300 when the gene was expressed (see Figure 5). In HeLa cells the proximal enhancer of IFNB1 has been reported to be completely nucleosome-free prior to induction [13], but we found that in primary BMDMs the corresponding region was lowly occupied by nucleosomes prior to gene expression and became completely nucleosome-free upon induction. Together, the changes in chromatin architecture at all the enhancers we have analyzed, both proximal and distal, were similar: enhancers were only moderately occupied by nucleosomes in resting macrophages and a central region was completely cleared of nucleosomes when the associated genes were induced. The size of the cleared region varied from about 1 nucleosome (at the proximal enhancer of IFNB1) to removal of 2–3 nucleosomes in the distal enhancers of IL12B, IFNB1 and IL1A (compare Figures 1A, 2A, 4A and C). The small size of the nucleosome-free region in the proximal enhancer of IFNB1 is in agreement with the assembly of an enhanceosome at this site, which forms a highly organized structure with a relatively small DNA-footprint [26]. Together, our data suggest that enhancers of pro-inflammatory genes undergo similar changes in nucleosome occupancy regardless of their distance from a TSS, and that clearance of enhancer nucleosomes is required to allow binding of *cis*-regulatory TFs. Moreover, we hypothesize that removal of nucleosomes at the promoter of IFNB1 may occur inadvertently due to its proximity to the proximal enhancer.

IL1A and IFNB1 have been classified as primary response genes while IL12B is a secondary response gene, and it has been shown that they differ in their induction kinetics as well as in their dependence on newly synthesized factors for efficient induction [50]. We find that nucleosome removal at the IL1A enhancer occurs with faster kinetics than at the IL12B enhancer (see Figure 2E) and we hypothesize that the different kinetics may indicate the involvement of different nucleosome remodelers as has been suggested [40]. While it is possible that nucleosomes may be removed from these regions by competition of signal-induced TFs for binding to their sites, the rapid kinetics we have observed strongly suggest that nucleosome remodelers are involved (see Figure 2E). Future studies will reveal, which remodelers play a role at these and other enhancers of inducible genes.

## Supporting Information

Table S1 **Student's tests were performed using normalized data from at least 3 (to 6) independent experiments performed with various antibodies as described in the legend of** **Figure 3** **.** All the measurements at the 2–4 locations in each enhancer or promoter, as well as the measurements at a single location in each ORF or in the IL12B intervening region were pooled from each experiment and Student's Tests (two-tailed, equal variance) were performed on each dataset. Table S1 shows the P-values obtained. We compared the significance of factor binding in resting BMDMs (0 h) versus B-cells, and in BMDMs after 1.5 h or 3 h LPS induction versus binding in resting BMDMs.
(TIFF)

Table S2 **Student's tests were performed as described in the legend for Table S1 using normalized data from at least 3 independent experiments performed with various antibodies as described in the legend of** **Fig. 5** **.** Table S2 shows the P-values obtained.
(TIFF)
